# Humoral Hypercalcemia of Malignancy with a Parathyroid Hormone-Related Peptide-Secreting Intrahepatic Cholangiocarcinoma Accompanied by a Gastric Cancer

**DOI:** 10.1155/2017/7012520

**Published:** 2017-05-10

**Authors:** Katsushi Takeda, Ryosuke Kimura, Nobuhiro Nishigaki, Shinya Sato, Asami Okamoto, Kumiko Watanabe, Sachie Yasui

**Affiliations:** ^1^Department of Endocrinology and Metabolism, Nagoya City West Medical Center, 1-1-1 Hirate-cho, Kita-ku, Nagoya 462-8508, Japan; ^2^Department of Gastroenterology, Nagoya City West Medical Center, 1-1-1 Hirate-cho, Kita-ku, Nagoya 462-8508, Japan; ^3^Department of Experimental Pathology and Tumor Biology, Nagoya City University Graduate School of Medical Sciences, 1 Kawasumi, Mizuho-cho, Mizuho-ku, Nagoya 467-8601, Japan

## Abstract

Humoral hypercalcemia of malignancy (HHM) is caused by the oversecretion of parathyroid hormone-related peptide (PTHrP) from malignant tumors. Although any tumor may cause HHM, that induced by intrahepatic cholangiocarcinoma (ICC) or gastric cancer (GC) is rare. We report here a 74-year-old male who displayed HHM with both ICC and GC and showed an elevated serum PTHrP level. Treatment of the hypercalcemia with saline, furosemide, elcatonin, and zoledronic acid corrected his serum calcium level and improved symptoms. Because treatment of ICC should precede that of GC, we chose chemotherapy with cisplatin (CDDP) and gemcitabine (GEM). Chemotherapy reduced the size of the ICC and decreased the serum PTHrP level. One year after diagnosis, the patient was alive in the face of a poor prognosis for an ICC that produced PTHrP. Immunohistochemical staining for PTHrP was positive for the ICC and negative for the GC, leading us to believe that the cause of the HHM was a PTHrP-secreting ICC. In conclusion, immunohistochemical staining for PTHrP may be useful in discovering the cause of HHM in the case of two cancers accompanied by an elevated serum PHTrP level. Chemotherapy with CDDP and GEM may be the most appropriate treatment for a PTHrP-secreting ICC.

## 1. Introduction

Hypercalcemia is a well-known complication of cancer as seen in the between 20 and 30 percent of cancer patients [1]. Malignant-associated hypercalcemia (MAH) is classified into four groups: humoral hypercalcemia of malignancy (HHM), local osteolytic hypercalcemia (LOH), excess 1,25(OH)_2_D secretion, and ectopic parathyroid hormone (PTH) secretion. HHM is associated with 80 percent of MAHs and is caused by the effects of the oversecretion of parathyroid hormone-related peptide (PTHrP) [1–3]. Although HHM can essentially be caused by any tumor [1], its induction by cholangiocarcinoma or gastric cancer (GC) is rare [4, 5]. In addition, to date, cases of HHM complicated by two cancers—cholangiocarcinoma and gastric cancer—have not been reported.

Herein, we report the first case of HHM induced by an intrahepatic cholangiocarcinoma (ICC) that secretes PTHrP, in conjunction with a GC identified by immunohistochemical staining.

## 2. Case Presentation

A 74-year-old male patient was admitted for hypercalcemia. Over the preceding two months he had suffered from a loss of appetite and had a history of seborrheic keratosis and hypertension. His weight was 47.4 kg, height 153.2 cm, temperature 36.6°C, heart rate 127 beats/minute, and blood pressure 143/92 mmHg. He had tenderness of the right hypochondrium, but an abdominal mass was not palpable.

Laboratory analyses revealed that the patient's corrected serum calcium level was elevated at 14.8 mg/dL. Serum carcinoembryonic antigen (CEA) and carbohydrate antigen (CA) 19-9 were within normal range. Serum alpha fetoprotein (AFP) showed a normal level of 3.8 ng/mL. The serum PTHrP level was elevated at 26.6 pmol/L and the serum intact PTH level was low at 9 pg/mL (Table 1).

**Figure 1:**
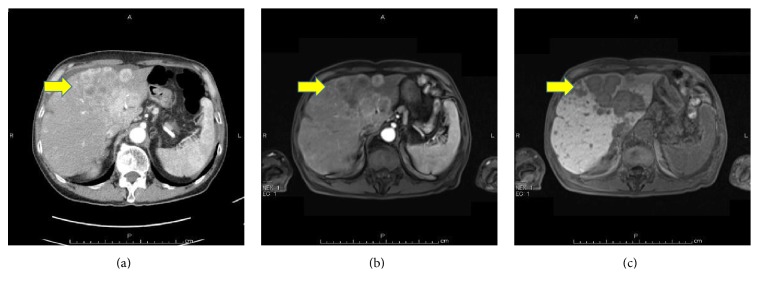
Dynamic contrast-enhanced computed tomography (CT) scans showed multiple masses in the patient's liver, suggesting intrahepatic cholangiocarcinoma (ICC; (a), arrow). His liver tumors showed an enhancement of the peripheral zone in the early phase ((b), arrow) and a low intake in the hepatobiliary phase ((c), arrow) in a gadolinium ethoxybenzyl diethylenetriamine pentaacetic acid- (Gd-EOB-DTPA-) enhanced magnetic resonance imaging (MRI).

**Figure 2:**
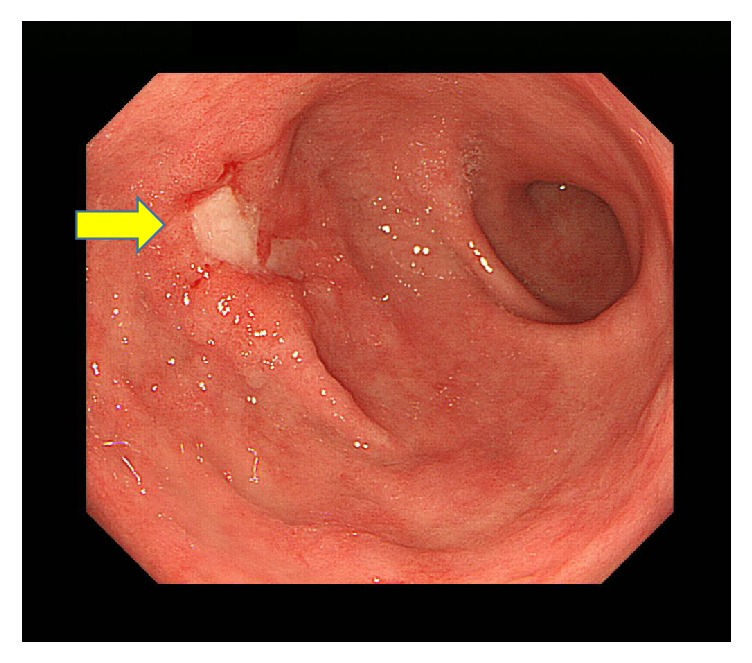
Esophagogastroduodenoscopy revealed an infiltrative ulcerative carcinoma (arrow) in the anterior wall of the antrum of the patient's stomach.

Dynamic contrast-enhanced computed tomography (CT) scans revealed a large mass, 76 mm in diameter, and multiple masses in the patient's liver. These masses showed an enhancement of the peripheral zone in the early phase of CT; the inside of such masses gradually became enhanced, suggesting an ICC with intrahepatic metastases (Figure 1(a)). Using fluorodeoxyglucose-positron emission tomography (FDG-PET), the patient's liver tumor showed a SUVmax 7.1 uptake value for FDG. Bone scintigraphy did not reveal any bone metastases. Magnetic resonance imaging (MRI) showed a slightly low signal intensity on a T1-weighted image and a slightly high signal intensity on a T2-weighted image. These tumors showed an enhancement of the peripheral zone in the early phase and low intake of gadolinium ethoxybenzyl diethylenetriamine pentaacetic acid- (Gd-EOB-DTPA-) enhanced MRI in the hepatobiliary phase (Figures 1(b) and 1(c)). Esophagogastroduodenoscopy revealed an infiltrative ulcerative carcinoma in the anterior wall of the antrum of the patient's stomach (Figure 2).

**Figure 3:**
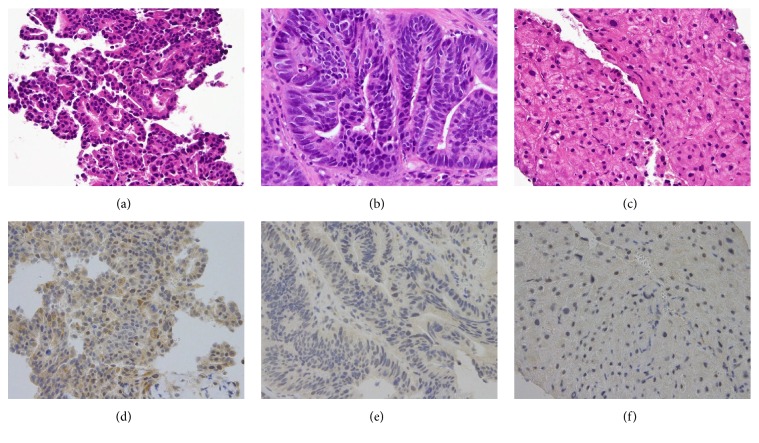
Histological examination of an ICC (a, d), gastric cancer (GC; (b, e)), and normal hepatocyte (c, f). Hematoxylin and eosin staining (×400) of an ICC (a), an adenocarcinoma suggesting GC (b), and normal hepatocyte (c). Immunohistochemical staining by anti-PTHrP antibody (H-137; Santa Cruz Biotechnology) in an ICC (d), GC (e), and normal hepatocyte (f).

A histological examination of a biopsy specimen from the hepatic tumor revealed an ICC (Figure 3(a)). Immunohistochemically, ICC tumor cells were positive for cytokeratin 7 and PTHrP (Figure 3(d)) and negative for cytokeratin 20, CDX2, and CA19-9. A histological examination of a biopsy specimen from the GC revealed an adenocarcinoma (Figure 3(b)). Immunohistochemically, GC tumor cells were positive for cytokeratin 7, cytokeratin 20, CDX2, and CA19-9 and were negative for PTHrP (Figure 3(e)). These findings indicated that this patient had two cancers, ICC and GC, and that his HHM was induced by the oversecretion of PTHrP from the ICC.

**Figure 4:**
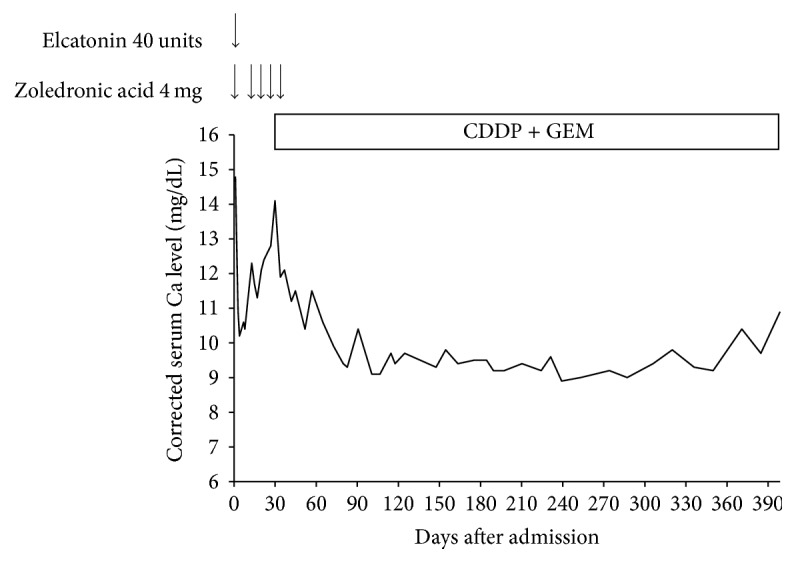
The corrected serum Ca level was stable without bisphosphonate after the patient commenced chemotherapy with cisplatin (CDDP) and gemcitabine (GEM).

After admission, the patient's hypercalcemia was treated with saline, furosemide, elcatonin, and zoledronic acid. His corrected serum calcium level and symptoms subsequently improved. We used elcatonin only a single time on admission and zoledronic acid five times during the five weeks after admission. Subsequently, his corrected calcium level was kept under 11 mg/dL without using elcatonin and bisphosphonate (Figure 4).

**Figure 5:**
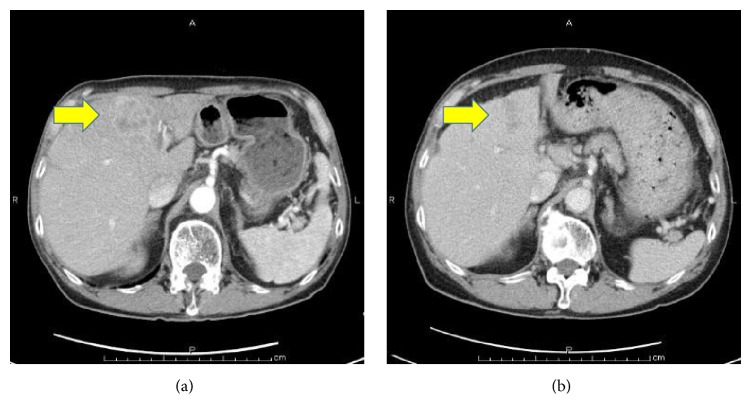
CT scans showed that the patient's liver tumors (arrows) were reduced by the 78th (a) and 238th day (b) of admission after chemotherapy with CDDP and GEM.

**Figure 6:**
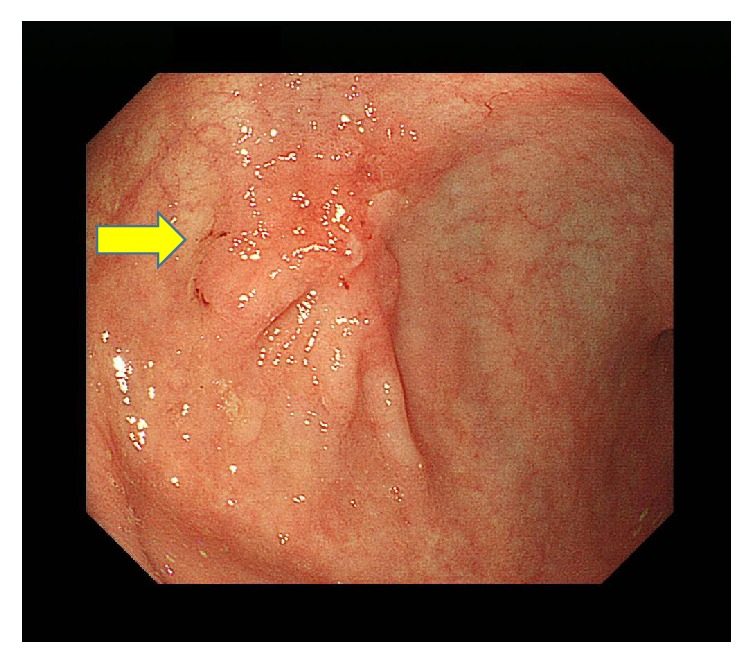
Esophagogastroduodenoscopy showed that the patient's gastric cancer (arrow) was reduced on the 273rd day.

The patient's prognosis was dependent on the ICC since a prognosis for this type of cancer is generally known to be poor and his ICC was complicated by intrahepatic metastasis. Therefore, we treated him with chemotherapy using cisplatin (CDDP) and gemcitabine (GEM). We started chemotherapy on the 30th day after admission, and he was discharged on the 39th day. On the 78th day, his liver tumors were reduced (Figure 5(a)). Moreover, on the 238th day, his liver tumors were smaller than tumors observed on the 78th day (Figure 5(b)). In addition, the volume of his gastric cancer was also decreased on the 273rd day (Figure 6), suggesting that chemotherapy with CDDP and GEM was also effective for his gastric cancer. His serum PTHrP level had improved to 4.9 pmol/L by the 101st day. A year after diagnosis, the patient was alive.

## 3. Discussion

HHM is usually caused by the oversecretion of PTHrP by a malignant tumor. Typical tumors causing HHM include various squamous cell carcinomas, renal cancer, ovarian cancer, endometrial cancer, human T-cell lymphotropic virus- (HTLV-) associated lymphoma and breast cancer [1]. The symptoms of HHM are often mild and nonspecific. Nevertheless, HHM is associated with substantial mortality, with about 50% of cancer patients who show hypercalcemia dying within 30 days [2, 6]. Therefore, the early treatment of HHM and control of the serum calcium level are important.

PTHrP has been purified from a human lung cancer cell line [7] and has significant sequence homology with the amino-terminal end of PTH [3, 7–9]. PTHrP is normally synthesized by various tissues and has important physiological roles. For example, in cartilage PTHrP regulates its proliferation and differentiation [10]. It is also produced in the placenta, where it regulates the fetal serum calcium level [11]. However, it is known as a factor responsible for HHM since it enhances the renal retention of calcium and increases bone resorption [1]. In addition, it has also been recognized as one of the causes of adipose browning and cachexia recently and new treatments of PTHrP may be developed to improve the prognosis of cancer patients in the future [12–14]

ICC accounts for 4.4 percent of primary liver cancers in Japan, so it is relatively rare [15]. ICC is frequently clinically silent in its early stages and is, therefore, often only diagnosed when it develops into an advanced cancer; this is a leading reason for its poor prognosis.

Cholangiocarcinoma is a malignant neoplasm arising from the biliary epithelium and can be anatomically classified into intrahepatic, perihilar, and distal extrahepatic tumors [16]. Surgical resection is the only curative treatment for cholangiocarcinoma; however, most patients with this tumor are not operative candidates [4, 16]. Chemotherapy for cholangiocarcinoma is administered to those patients who are not operative and results have been largely disappointing, especially for cholangiocarcinoma that produces PTHrP [4, 16]. To the best of our knowledge, patients diagnosed with a PTHrP-secreting cholangiocarcinoma who lived for more than six months have been reported in only four instances, including the present case (Table 2) [4, 17, 18]; chemotherapy was performed for each case but surgical resection was not. This suggests the possibility that chemotherapy is one of the effective treatments for a PTHrP-secreting cholangiocarcinoma, like a non-PTHrP-secreting cholangiocarcinoma. However, further reports are needed in order to decide the best therapy for these types of tumors.

Immunohistochemical staining is a widely used technique that demonstrates the expression and distribution of a specific antigen by antigen-antibody immunoreaction. It is often used not only in research, but also in clinical practice, for example, in pathological diagnosis and the determination of molecular-targeted therapy. However, immunohistochemical staining for PTHrP is still uncommon. There are opinions that antibodies for PTHrP may be considered nonspecific. However, an antibody for PTHrP (H-137; Santa Cruz Biotechnology) is negative for normal hepatocyte which we were able to get when we performed the biopsy (Figure 3(f)), suggesting that this antibody has the specificity for PTHrP. Therefore, our case suggests that immunohistochemical staining by an anti-PTHrP antibody may be useful in the search for the cause of HHM in the case of two cancers accompanied by an elevated serum PTHrP level. Because of an aging society, the occurrence of two cancers together is no longer rare. Therefore, the incidence of HHM with two cancers is also expected to increase. The identification of a cancer secreting PTHrP by using immunohistochemical staining will allow the correct prediction of fluctuation of serum calcium level.

In conclusion, we have reported the first case of a patient with a PTHrP-secreting ICC accompanied by GC.

## Figures and Tables

**Table 1:** Laboratory data on admission.

WBC	8900/*μ*L
RBC	476 × 10^4^/*μ*L
Hb	15.9 g/dL
PLT	21.7 × 10^4^/*μ*L
TP	6.7 g/dL
Alb	3.6 g/dL
T-Bil.	0.6 mg/dL
D-Bil.	0.2 mg/dL
ALP	515 U/L
GOT	64 U/L
GPT	26 U/L
LDH	190 U/L
Amy	57 U/L
CPK	27 U/L
UA	10.0 mg/dL
BUN	28.5 mg/dL
CRN	1.27 mg/dL
Na	138 mmol/L
K	3.8 mmol/L
Cl	98 mmol/L
Ca	14.4 mg/dL
Corrected Ca	14.8 mg/dL
I-P	2.8 mg/dL
Mg	1.7 mg/dL
CRP	1.5 mg/dL
Glu	129 mg/dL
CEA	1.5 ng/mL (0.0~5.0)^*∗*^
CA19-9	6.2 U/mL (0.0~37.0)^*∗*^
AFP	3.8 ng/mL (0.0~10.0)^*∗*^
HBs antigen	(—)
HCV antibody	(—)
PTH intact	9 pg/mL (10~65)^*∗*^
PTHrP	26.6 pmol/L (0~1.1)^*∗*^

^*∗*^Numbers in parentheses indicate the normal range; WBC: white blood cells; RBC: red blood cells; Hb: hemoglobin; PLT: platelets; TP: total protein; Alb: albumin; T-Bil.: total bilirubin; D-Bil.: direct bilirubin; ALP: alkaline phosphatase; GOT: glutamic oxaloacetic transaminase; GPT: glutamic pyruvic transaminase; LDH: lactate dehydrogenase; Amy: amylase; CPK: creatine phosphokinase; UA: uric acid; BUN: blood urea nitrogen; CRN: creatinine; Na: sodium; K: potassium; Cl: chlorine; Ca: calcium; I-P: inorganic phosphate; Mg: magnesium; CRP: C-reactive protein; Glu: glucose; CEA: carcinoembryonic antigen; CA: carbohydrate antigen; AFP: alpha fetoprotein; HBs: hepatitis B surface; HCV: hepatitis C virus; PTH: parathyroid hormone; PTHrP: parathyroid hormone-related peptide.

**Table 2:** Reported cases of cholangiocarcinoma secreting PTHrP who are alive for more than half a year.

Number	Authors	Age	Reported year	Sex	Ca level	PTHrP	Therapy	Prognosis
(1)	Davis et al. [17]	54	1994	Male	16.4	5.2	FUDR + 5-FU	Survival (for 6 months)
(2)	Yamada et al. [4]	43	2009	Male	14.4	5.0	TAE + PTPE + GEM + S-1 + Radiation	Died (after 14 months)
(3)	Lim et al. [18]	63	2013	Male	^*◎*^12.1	^*※*^6.7	CAP + CDDP + Radiation, second-line GEM	Died (after almost 1 year)
(4)	Our case	74	2016	Male	^*◎*^14.8	26.6	CDDP + GEM	Survival (after 1 year)

FUDR: floxuridine; 5-FU: 5-fluorouracil; GEM: gemcitabine; CAP: capecitabine; CDDP: cisplatin; TAE: transcatheter arterial embolization; PTPE: percutaneous transhepatic portal embolization; ^*※*^PTHrP reported by Lim et al. was measured 9 months after an ICC diagnosis; ^*◎*^Ca level means corrected Ca.
